# Effect of adipocytes on the function and activity of T cells in tumor microenvironment

**DOI:** 10.3389/fimmu.2025.1688342

**Published:** 2025-10-22

**Authors:** Lujia Zhu, Shujun Xu, Yuhao Ye, Yang Xiong, Qiushuang Li

**Affiliations:** ^1^ Department of Pharmacy, Affiliated Hospital of Shaoxing University, Shaoxing, China; ^2^ School of Pharmaceutical Sciences, Zhejiang Chinese Medical University, Hangzhou, China; ^3^ Science Research Department, The First Affiliated Hospital of Zhejiang Chinese Medical University (Zhejiang Provincial Hospital of Chinese Medicine), Hangzhou, China

**Keywords:** tumor microenvironment, cancer-associated adipocyte, immunity, T cell, inflammatory factors

## Abstract

The tumor microenvironment (TME) comprises non-cancerous cells, extracellular matrix, and signaling molecules that interact with tumor cells. These dynamic interactions critically influence tumor development, progression, metastasis, and treatment response. Cancer-associated adipocytes (CAAs), as a main component of the tumor-adipose microenvironment (TAME), have various functions, including remodeling the extracellular matrix and interacting with tumor cells or infiltrated leukocytes through a variety of mutual signals. Dysfunctional adipocytes can release different metabolic substrates, adipokines and cytokines to affect the activity and function of immune cells in TME, especially T cells, thus promoting the proliferation, progression, invasion and migration of cancer cells. In this review, we summarize the effects of secretions of adipocytes on the activity and function of different types of T cells in TME, and discuss the possible targets of adipocytes in cancer therapy to provide new ideas for anti-cancer therapy by targeting adipocytes.

## 1 Introduction

The tumor microenvironment (TME), comprising cancer cells, stromal cells, and extracellular matrix (ECM), significantly influences immune surveillance and response to anti-cancer therapies (1, 2). Adipocytes are key stromal components within the TME. Interactions between tumor cells and neighboring adipocytes significantly shape the local milieu. The TME harbors various stromal cells (fibroblasts, adipocytes, preadipocytes, endothelial cells) and immune cells (NK cells, macrophages (M1, M2), dendritic cells, T cells, B cells) (3, 4). Emerging research highlights the complex interplay between adipocytes and T cells. Adipocyte-derived factors-including metabolic substrates, adipokines, and cytokines–exert profound effects on T cell activity and function, contributing to cancer progression (5–7).

Adipocytes, once viewed merely as energy storage depots, are now recognized as dynamic regulators within the TME. Understanding how adipocyte-derived signals modulate immune cell function, especially T cells, opens avenues for novel therapeutic strategies. Targeting adipocyte factors that suppress T-cell activity or promote immunosuppressive phenotypes could restore immune surveillance and enhance anti-tumor immunity (8, 9).

Within the TME, adipocytes fuel tumor progression by providing fatty acids (FAs) as an energy source. They also promote metastasis and invasion, processes involving epithelial-mesenchymal transition (EMT), ECM remodeling, and colonization. Adipocytes induce EMT markers and secrete matrix metalloproteinases (MMPs) that degrade the ECM (10–13). Alarmingly, adipocytes can metabolize anti-cancer drugs into less active forms, reducing efficacy (14).

T cells, especially CD8^+^ cytotoxic T lymphocytes (CTLs), are pivotal anti-tumor effectors. CTLs recognize tumor antigens via T-cell receptor (TCR)-MHC binding, forming an immunological synapse. They eliminate targets through FAS/FASL engagement and perforin/granzyme release (15). However, TME immunosuppression, driven by factors from cancer-associated fibroblasts (CAFs), regulatory T cells (Tregs), and M2 macrophages, can impair CTL function and induce exhaustion (16–20). CD4^+^ T cells exhibit diverse functions: Th1 exerts anti-tumor effects while Th2, Th17 (context-dependent), and Tregs contribute to immunosuppression. Tregs suppress immunity via CTLA-4/CD80/CD86 binding, IL-2 consumption, inhibitory cytokines, and direct cytotoxicity (21). Modulating T-cell function is thus crucial for cancer therapy.

Over the years, significant advancements have been made in elucidating the intricate relationship between obesity, adipocytes, and cancer. Elevated levels of adipokines such as estrogens, along with metabolic substrates including FAs, cholesterols, and exosomes, are believed to drive cancer progression by impairing T cell-mediated immune responses within TME. Additionally, related cytokines such as CCL-2, interleukins (IL), and tumor necrosis factor-alpha (TNF-α) further contribute to the immunosuppressive milieu in the TME.

This review consolidates current knowledge on how adipocytes modulate T cell function and activity, explores underlying mechanisms, and identifies potential therapeutic targets on adipocytes for cancer treatment.

## 2 Adipocytes secrete inflammatory factors that modify the function and activity of T cells

### 2.1 Via metabolic substrates

#### 2.1.1 Via FAs

FAs secreted by adipocytes constitute a significant component of the lipid-rich TME and play a multifaceted role in modulating T cell function and anti-tumor immunity. When hungry, adipocytes break down triglycerides into fatty acids and secrete them (22). While FAs serve as essential metabolic substrates for T cells, their abundance in the TME can profoundly influence T cell metabolism and effector functions. The increased availability of FAs within the TME has been associated with alterations in T cell metabolism, particularly affecting CTLs. Despite their reliance on glycolysis as the primary metabolic pathway upon activation, CD8^+^ T cells can catabolize FAs via fatty acid oxidation (FAO). However, the preferential engagement of FAO over glycolysis in activated CD8^+^ T cells diminishes their effector function, ultimately impairing their anti-tumor activity (23, 24). Intriguingly, this metabolic shift toward FAO prolongs the survival of memory CD8^+^ T cells, underscoring the intricate balance between fatty acid metabolism and T cell fate determination (25, 26).

Recent studies have provided compelling evidence linking dysregulated lipid metabolism in adipocyte-rich environments, such as obesity, to the promotion of T cell dysfunction and tumor progression. Specifically, the upregulation of carnitine palmitoyltransferase 1A (Cpt1a), a pivotal enzyme involved in FAO, has been identified as a key factor in obesity-induced T cell dysfunction and the subsequent enhancement of tumor growth (27). Furthermore, lipid accumulation within the TME has been shown to impair the function of CD8^+^ T cells through alterations in fatty acid uptake and metabolism. This dysregulation manifests as increased uptake of long-chain FAs and reduced activity of very-long-chain acyl-CoA dehydrogenase (VLCAD) in CD8^+^ T cells, resulting in intracellular lipid accumulation, mitochondrial dysfunction, and compromised effector responses (28). These findings underscore the critical role of lipid metabolism in shaping the immune landscape within the TME and highlight potential therapeutic targets for restoring T cell function and enhancing anti-tumor immunity.

Some preclinical studies have provided valuable insights into the potential role of omega-3 fatty acids (N-3 FAs), particularly in the context of cancer immunotherapy (29). Specifically, supplementation with N-3 FAs has been found to mitigate breast tumor growth by modulating the TME (30). These studies have demonstrated that N-3 FAs promote the infiltration of CD3^+^ T cells into the TME, thereby enhancing the anti-tumor immune response (31). Additionally, N-3 FAs have been shown to augment the anti-inflammatory activity of interleukin-10 (IL-10), a cytokine known for its immunosuppressive properties. These findings highlight the complex interplay between lipid metabolism, immune function, and cancer progression (32). Targeting adipocyte-derived FAs, such as N-3 FAs, represents a promising avenue for cancer immunotherapy, offering potential therapeutic benefits in modulating the immune response against cancer cells.

#### 2.1.2 Via cholesterol

Cholesterol, a prominent secretion of adipocytes and a crucial component of cell membranes, has emerged as a significant factor in the clinical progression of breast cancer (33). Serum cholesterol comprises various fractions, including total cholesterol, high-density lipoprotein (HDL), low-density lipoprotein (LDL), and very-low-density lipoprotein (VLDL). Notably, total cholesterol, LDL, and the oxysterol metabolite 27-hydroxycholesterol (27-HC) have been implicated in regulating T cell function within the TME.

Ma et al. has elucidated that cholesterol in the TME upregulates the expression of immune checkpoints programmed cell death protein 1 (PD-1) and 2B4 by inducing endoplasmic reticulum stress in CD8^+^ T cells, leading to T cell exhaustion (34). Additionally, studies have indicated that LDL can be internalized by Vγ9Vδ2 T cells, a subtype of effector T cells, resulting in reduced expression of key activation markers such as IFN-γ, NKG2D, and DNAM-1, thereby dampening T cell activation and function (35). Consequently, impaired immune surveillance may facilitate tumor evasion and diminish patient survival rates, particularly in cases of hypercholesterolemia. However, further prospective clinical trials are required to validate the association between LDL levels and cancer patient survival rates.

Furthermore, 27-HC, an oxysterol derivative of cholesterol, has been implicated in inhibiting the activity of CD8^+^ cytotoxic T cells by suppressing the activation and recruitment of neutrophils and γδ-T cells, consequently promoting breast cancer metastasis (36). Recent investigations have shed light on the underlying mechanism, revealing that 27-HC acts on myeloid cells in an Liver X receptor (LXR)-dependent manner, thereby impairing T cell proliferation and cytotoxicity. This was corroborated by myeloid-specific knockout of the CYP27A1 gene, which resulted in reduced metastatic breast cancer in murine models (37). These findings underscore the multifaceted role of cholesterol and its derivatives in modulating immune responses within the TME, highlighting potential therapeutic avenues for disrupting cholesterol-mediated immunosuppression in cancer.

#### 2.1.3 Via exosomes

As potent mediators of intercellular communication, exosomes derived from adipocytes harbor a diverse cargo of lipids, proteins, and microRNAs (miRNAs), which play pivotal roles in various biological processes, including immune modulation. It can regulate the physiological functions of different tissues and organs in autocrine, endocrine and paracrine ways (38).

Research by Fan et al. has revealed intriguing insights into the role of miRNAs in adipocyte-derived exosomes in lung adenocarcinoma. Specifically, they found that downregulation of miR-27a-3p in exosomes derived from adipocytes inhibits the proliferation of CD4^+^ T cells and the secretion of IFN-γ*in vitro (* 39). In addition to miRNA, lncRNA also plays a role in it. LINC01119 is a differentially expressed lncRNA in ovarian cancer (OC). LINC01119 encapsulated by CAAs-derived exosomes promoted M2 polarization of macrophages, thereby inhibiting the proliferation of CD3^+^ T cells (40). What’s more, adipose-derived stem cell exosomes (ASC-exos) could be taken up by CD4^+^ T cells, thus inhibiting Th 1 and Th 17 differentiation and promoting Tregs differentiation. Through these effects, ASC-exos promoted breast cancer characterization and TME immunosuppression (41). Moreover, extracellular vesicles (EV), similar to exosomes, could mediate tumorigenesis. In OC, CAAs-EV delivered sirtuin 1(SIRT1) to OC cells. SIRT1 transcriptionally activated the expression of CD24, which up-regulated the expression of Siglec-10. Finally, the up-regulation of Siglec-10 promoted the apoptosis of CD8^+^ T cells, thereby promoting tumorigenesis in mice (42). These findings underscore the intricate interplay between adipocyte-derived exosomes and T cell function in the TME, shedding light on potential therapeutic targets for immune modulation in cancer therapy. In summary, adipocytes metabolic substrates have significant effects on T cells, and how them affect T cells through metabolic substrates is summarized in **Table 1**.

**Table 1:** Adipocytes modify the function and activity of T cells via metabolic substrates.

Metabolic substrates	Target cell	Effect on target	Key molecules/pathways	Reference
Fatty acid	CD8^+^ T cell	Decrease function	VLCAD↓, Cpt1a↑, FAO↑	(23)
Cholesterol	CD8^+^ T cell	Decrease function	PD-1↑, 2B4↑	(34, 37)
Exosome (miR-27a-3p)	CD4^+^ T cell	Decrease function	N/A	(39)
Exosome (LINC01119)	CD3^+^ T cell	Decrease function	M2 polarization↑	(40)
ASC-exos	CD4^+^ T cell, Tregs	Increase function	Th1↓, Th17↓	(41)
CAAs-EV	CD8^+^ T cell	Decrease function	SIRT1, Siglec-10↑	(42)

### 2.2 Via released adipokines

#### 2.2.1 Via leptin

As a pro-inflammatory cytokine, leptin has been implicated in cancer progression (43, 44). The level of leptin in the circulation fluctuates day and night, and more is secreted at night (45). In the TME, cancer cells exert intricate control over adipocyte differentiation, inhibiting the maturation of preadipocytes while promoting their differentiation into adipocytes (46). Leptin, whose mRNA is absent in preadipocytes, becomes detectable upon their differentiation into mature adipocytes. It has been reported that dietary fructose initiated adipocytes to produce leptin in a mTORC 1-dependent manner, and mediated leptin to enhance the anti-tumor function of CD8^+^ T cells, which provided a reference for inhibiting tumors through leptin (6, 47). Upon binding to its receptor, leptin activates downstream signaling pathways involving JAK2/STAT3, ERK1/2, AP1, PI3K, and MAPK (44, 48–50).

STAT3, a pivotal transcription factor involved in tumor cell survival and proliferation, also mediates tumor-promoting inflammation (51). Its role extends to the regulation of various T cell subsets, including Th17 cells, CD4^+^ follicular helper cells, Tregs, and CD8^+^ effector or memory T cells. Interestingly, studies have identified STAT3 binding sites in the promoter region of PD-1, a key inhibitory receptor on T cells (52). Wang et al. elucidated the mechanism underlying leptin-induced T cell dysfunction, linking increased PD-1 expression to upregulated phosphorylated STAT3 (pSTAT3), a hallmark of STAT3 activation. Furthermore, they demonstrated that leptin upregulates the expression of hepatitis A virus cellular receptor 2 (HAVCR2 or Tim3) and Cpt1a, thereby inhibiting the proliferation of PD-1^+^ CD8^+^ T cells (53).

Moreover, Zhang et al. uncovered the role of leptin in driving STAT3 activation and FAO in CD8^+^ T cells, resulting in suppressed glycolysis and effector function, consequently promoting the progression of obesity-related breast tumors (54). While leptin has been shown to impact various T cell subsets, including Tregs, it has also been reported to enhance the activity of naïve and memory T cells (55, 56).Interestingly, in the context of oncolytic virus therapy for melanoma, leptin’s overexpression has been associated with increased activation of CD8^+^ T cells and enhanced mitochondrial biogenesis. However, this beneficial effect of leptin appears to be diminished in obese mice, possibly due to leptin resistance in the setting of obesity (57).

#### 2.2.2 Via neuregulin 4

In addition, there is another adipokine which is called NRG4. Zhang et al. found the inhibitory effect of NRG4 on liver cancer. During diet-induced nonalcoholic steatohepatitis (NASH), NASH-associated macrophages induction and T cell depletion occurred before any obvious tumor in the liver, which increased the possibility that these immune disorders promoted the immunosuppressive liver microenvironment prone to cancer. NRG4, as an adipose endocrine factor, could inhibit the expansion of macrophages with tumor-associated macrophage molecular characteristics and inhibit the depletion of CD8^+^ T cells caused by tumor-associated macrophages, thereby exerting anti-hepatocellular carcinoma effects (58). Finally, the effects of adipocytes released adipokines on T cells are summarized in **Table 2**.

**Table 2:** Adipocytes modify the function and activity of T cells via released hormones.

Metabolic substrates	Target cell	Effect on target	Signal molecules involved	Reference
Leptin	CD8^+^ T cell;	Decrease function	STAT3↑, HAVCR2↑, Tim3↑, Cpt1a↑	(53, 54)
Neuregulin 4	CD8^+^ T cell	Increase function	N/A	(58)

### 2.3 Via released cytokines

#### 2.3.1 Via TNF-α

TNF-α is a crucial inflammatory mediator within the TME, originating from both tumor cells and stromal components, including adipocytes. This multifaceted cytokine participates in various cellular signaling pathways upon binding to its receptors, exerting profound effects on inflammation and cancer development (59). Notably, TNF-α has been implicated in augmenting the activity of TNFR2^+^ Tregs (60). In murine models of colon cancer, TNFR2 has been shown to promote the expansion of Tregs, thereby facilitating tumor metastasis (61). Similar observations have been documented in models of colorectal and liver cancer, where the blockade of TNFR2 effectively abrogates tumor-induced Treg amplification. Moreover, pre-treatment of Tregs with TNF-α prior to adoptive transfer enhances their suppressive capacity against anti-tumor immunity (62). These findings underscore the intricate role of TNF-α in shaping the immunosuppressive milieu of the TME and its potential as a therapeutic target in cancer immunotherapy.

#### 2.3.2 Via interleukins

ILs, including IL-6, IL-8, and IL-10, among others, are intimately associated with cancer progression (63, 64). Notably, IL-6 and IL-10 have been shown to prominently induce T cell dysfunction, with IL-6 particularly implicated in poor tumor prognosis (65). Co-culture experiments involving adipocytes and breast cancer cells have revealed heightened expression and secretion of IL-6 by adipocytes, thereby fostering cancer cell invasion and migration (66, 67). IL-6 can impede Thelper1 (Th1) differentiation, thereby impairing the activation of Th1-mediated CTLs (68). Moreover, IL-6 has been demonstrated to modulate the production of IL-10 and vascular endothelial growth factor (VEGF), further inhibiting CTL function (69). The mechanism underlying CD8^+^ T cell exhaustion induced by IL-6 may involve the activation of the STAT3 pathway via NF-κB and IL-6-GP130-Janus kinase (JAK) signaling pathways, thereby exerting inhibitory effects on tumor progression (70). These insights underscore the pivotal role of ILs in shaping the immunosuppressive landscape of the TME and their potential as therapeutic targets in cancer management.

#### 2.3.3 Via chemokines

Chemokines play a pivotal role in the recruitment and activation of leukocytes, including T cells, natural killer cells, and monocytes, within the TME. Chemokines such as CCL2, CCL5, CCL4, and CXCL8 have been implicated in the initiation and progression of cancer (71). Specifically, in the TME, various cell types, including cancer cells, fibroblasts, tumor-infiltrating monocytes, adipocytes, and endothelial cells, contribute to the production and secretion of CCL2 (72). Studies by Santander et al. have demonstrated that co-culturing breast tumor cells with adipocytes results in increased expression of CCL2, leading to enhanced recruitment of monocytes/macrophages to the tumor site (73). Moreover, CCL2 has been shown to promote the polarization of tumor-associated macrophages (TAMs), which exert immunosuppressive effects by inhibiting T cell activation and fostering angiogenesis (74). These findings underscore the pivotal role of chemokines in orchestrating the immune landscape within the TME and highlight their potential as therapeutic targets in cancer intervention strategies.

#### 2.3.4 Via estrogen

Estrogen, a key hormone, plays a pivotal role in driving the progression of breast cancer through its interaction with three receptors: estrogen receptor α (ERα), estrogen receptor β (ERβ), and G-protein coupled estrogen receptor 1 (GPER-1), as well as estrogen-related receptors (ERRs). ERα, a ligand-activated transcription factor, serves as the primary driver in approximately 70% of breast cancer cases (75). Conversely, ERβ, encoded by the ESR2 gene, is the most abundantly expressed estrogen receptor in normal mammary glands and has shown conflicting roles in breast cancer, with some *in vitro* studies suggesting its inhibitory effects on cancer cell progression and invasion (76, 77). GPER-1, a transmembrane protein, operates independently of ERα and ERβ and has been implicated in estrogen-mediated signaling pathways in breast cancer (78). Additionally, ERRs, constitutively active orphan receptors, modulate estrogen responses in breast cancer cells despite not directly binding to estrogen molecules (79). Furthermore, both ERα and ERβ are expressed in T cells. In lung and cervical tumor samples, Erα signaling is associated with reduced infiltration of CD4^+^ and CD8^+^ T cells into the tumor microenvironment (80, 81). In the mouse breast cancer model, the mutation of ERb reduced the infiltration of CD4^+^ and CD8^+^ T cells and the concentration of IFN-γ in the tumor microenvironment, resulting in an increase in tumor volume (82). ^1^

Estrogen promotes breast cancer proliferation through the ER-membrane pathway, notably involving the MAPK/ERK signaling cascade (83). Inhibition of the MAPK pathway has been shown to alleviate local immunosuppression within the TME, consequently enhancing the infiltration of tumor-infiltrating lymphocytes (TILs) (84). The mechanism is related to the senescence of T cells, induction of ataxia-telangiectasia mutated protein-associated DNA damage is the cause for T cell senescence induced by both mouse tumor cells and Treg cells, which is also regulated by MAPK signaling (84). Moreover, heightened expression of estrogen receptors (ERs) has been associated with diminished infiltration of CD8^+^ T cells and reduced expression of PD-1/PD-L1 in breast cancer cells by suppressing IL-17 signaling and impeding Th17 cell infiltration (85). These insights underscore the multifaceted effects of estrogen signaling on breast cancer progression and immune modulation within the TME.

In addition, estrogen has direct immunomodulatory effects (86), ERβ signaling in CD8^+^ T cells boosts T cell receptor activation and antitumor immunity through a phosphotyrosine switch (82).

It is worth noting that adipocytes are not the main source of estrogen synthesis in the bodys, but in postmenopausal women, adipocytes become the main estrogen synthesis cells, leading to differences in the effects of estrogen on female cancer (87). Meanwhile, because men and women have different sex hormones, the differences can affect the response of male and female cancer patients to immunotherapy (88).

#### 2.3.5 Via adiponectin

Adipocyte-derived circulating protein adiponectin is a representative adipocytokine with two unique characteristics: its circulating concentration is about 3–6 orders of magnitude higher than that of common hormones and cytokines; although it is specifically produced by adipocytes, its concentration is inversely proportional to body fat mass (89). The tumor inhibitory effect of adiponectin is related to ARG1, it can inhibit the down-regulation of ARG1 in inflammatory tissues, thereby restoring T cell viability (89). Furthermore, adiponectin disrupted breast cancer cell metabolism by downregulating sterol regulatory element-binding protein 1 (SREBP-1) and fatty acid synthase (FAS), thereby promoting lipolysis and fatty acid oxidation while impairing lipid raft integrity. These metabolic effects, mediated through SIRT1 activation, were further confirmed *in vivo (* 90). All in all, adipocytes released cytokines have effects on T cells and the specific content is summarized in **Table 3**.

**Table 3:** Adipocytes modify the function and activity of T cells via released cytokines^2^.

Metabolic substrates	Target cell	Effect on target	Signal molecules involved	Reference
TNF-α	Treg	Increase function	TNFR2	(62)
IL-6, IL-10	CD8^+^ T cell	Decrease function	VEGF, STAT3	(69, 70)
CCL2, CCL5	CD8^+^ T cell	Decrease function	TAMs	(73, 91)
Estrogen	CD8^+^ T cell	Decrease function	MAPK, IL-17↓	(85, 92)
Adiponectin	CD8^+^ T cell and CD4^+^ T cell	Increase function	ARG1↑	(89)

TNF-α, tumor necrosis factor-alpha; Treg, regulatory T cells; IL-6, interleukins-6; IL-10, interleukins-10; VEGF, vascular endothelial growth factor; TAMs, tumor-associated macrophages; IL-17, interleukins-7.

### 2.4 Via other mechanisms

PD-1 is considered to be the main immune checkpoint for regulating tumor immunity in recent years. Many inflammatory factors and some metabolites can induce the expression of PD-1 on T cells (93). In TME, many types of cells express PD-1 ligand PD-L1, and adipocytes are no exception (94, 95). It is considered that mature adipocytes express higher level of PD-L1 than preadipocytes. Wu et al. found that highly expressed PD-L1 on mature adipocytes can prevent anti-PD-L1 antibodies from activating the anti-tumor function of CD8^+^ T cells *in vitro*. Treatment of adipocytes with inhibitors of adipogenic key transcription factor PPAR γ can reduce the expression of PD-L1 and restore the anti-tumor function of CD8^+^ T cells (96). In summary, these studies demonstrate the inhibitory effect of PD-L1 expression in adipocytes on T cell activity.

The adipocyte niche refers to the complex local microenvironment in which adipocytes are located (97). This microenvironment consists of a variety of different cell types, extracellular matrices, signaling molecules (such as hormones, cytokines), and neurovascular networks. According to experimental verification, T cells exposed to adipocyte niches showed impaired force transfer to TCRs-antigen complexes, which means that adipocyte-derived factors alter the cytoskeleton and mechanics of T cell receptor signaling (97). These results were verified in diet-induced obese mice. All in all, adipocyte niche leads to T cell dysfunction through cytoskeletal regulation and reduces TCR triggering by inhibiting TCR force (97). High levels of leptin in the obesity microenvironment increase the expression of PD1 receptors and the depletion of T cells, which may be harmful to the immunotherapy based on Chimeric Antigen Receptor T-cells (Car-T cells) and bispecific T-cell engagers (BiTE) (53).

Insulin resistance (IR), commonly observed in obese individuals, has been implicated in various diseases, including breast cancer (98). Epidemiological studies have highlighted a correlation between IR and breast cancer incidence. Insulin plays a multifaceted role in the differentiation and function of CD8^+^ T cells, while also influencing the secretion of adipocytokines (99). Moreover, insulin-like growth factor-I receptors (IGF-IRs) are prominently expressed in most subtypes of breast cancer and are integral components of key pathways driving tumor growth (100). Furthermore, insulin-like growth factor binding protein-3 (IGFBP-3) has been implicated in promoting breast cancer growth in immune-tolerant mouse models by inhibiting T cell infiltration into the TME (101). These findings underscore the complex interplay between insulin signaling, immune function, and cancer progression, shedding light on potential therapeutic targets for breast cancer management.

## 3 Targeting adipocytes in TME for drug development

Targeting adipocytes within the TME represents a promising avenue for cancer therapy, given their significant role in tumor progression and immune modulation. Adipocytes contribute to cancer progression through various mechanisms, including the secretion of adipokines, cytokines, and lipids, which collectively create an immunosuppressive and pro-tumorigenic microenvironment.

One approach to targeting adipocytes in cancer therapy involves modulating the signaling pathways involved in adipocyte-derived factors. For example, inhibiting the expression or activity of adipokines such as leptin or adiponectin, which have been implicated in promoting tumor growth and metastasis, could potentially hinder tumor progression. Similarly, targeting the secretion of pro-inflammatory cytokines like TNF-α or IL-6 from adipocytes may alleviate inflammation within the TME and enhance anti-tumor immune responses.

Another strategy is to interfere with lipid metabolism in adipocytes, as dysregulated lipid metabolism can fuel tumor growth and impair immune function. Inhibiting key enzymes involved in fatty acid synthesis or oxidation pathways, such as Cpt1a, may disrupt the energy supply to cancer cells and restore T cell function within the TME (27). Furthermore, targeting the crosstalk between adipocytes and immune cells, particularly T cells, holds promise for cancer immunotherapy. Adipocyte-derived factors can directly modulate T cell function and promote immune evasion by upregulating immune checkpoint molecules like PD-L1 on adipocytes. Strategies aimed at blocking these interactions, such as using antibodies against PD-L1 or targeting downstream signaling pathways involved in immune checkpoint regulation, could enhance T cell-mediated anti-tumor immune responses (85, 95). Moreover, emerging evidence suggests that adipocytes can influence the response to conventional cancer therapies, including chemotherapy and immunotherapy (102, 103). Understanding the interplay between adipocytes and therapeutic agents may provide insights into optimizing treatment efficacy and overcoming resistance mechanisms. Despite these potential therapeutic strategies, several challenges remain. Developing selective and efficient delivery systems to target adipocytes specifically within the TME while minimizing off-target effects on healthy adipose tissue is critical. Additionally, unraveling the complex signaling networks and metabolic pathways involved in adipocyte-cancer cell interactions requires further investigation to identify novel therapeutic targets.

In conclusion, targeting adipocytes in the TME represents a promising approach for cancer therapy. By disrupting adipocyte-derived signals, modulating lipid metabolism, and interfering with adipocyte-immune cell crosstalk, novel therapeutic interventions may hold the potential to improve treatment outcomes and overcome therapeutic resistance in cancer patients. Continued research efforts in this field are essential to translate these findings into clinical applications and ultimately benefit cancer patients.

## 4 Conclusions

Indeed, adipocytes play a multifaceted role in shaping the TME and modulating immune responses, particularly those mediated by T cells. Through the release of various metabolic substrates, adipokines, and cytokines, adipocytes can exert both direct and indirect effects on the function and activity of T cells within the tumor milieu.

FAs and cholesterol, two prominent metabolic substrates released by adipocytes, have been shown to impair the function of CD8^+^ effector T cells, thus compromising their ability to mount effective anti-tumor responses. Additionally, adipocyte-derived factors such as exosomes, leptin, insulin, and estrogen have been implicated in inhibiting the function and activity of CD8^+^ T cells, further contributing to the establishment of an immunosuppressive TME.

Furthermore, adipocytes can promote the function of Tregs through the action of factors like TNF-α and leptin. By enhancing the suppressive activity of Tregs, adipocytes facilitate immune evasion and tumor progression, highlighting the intricate interplay between adipocyte-derived signals and immune cell function in cancer. However, the precise mechanisms underlying adipocyte-mediated modulation of T cell metabolism, activation, and function remain incompletely understood, necessitating further investigation into this complex interplay. Additionally, the diverse array of secretions from adipocytes and the heterogeneity of T cell populations within the TME contribute to the complexity of this interaction, warranting comprehensive studies to elucidate the underlying mechanisms. Moreover, given the widespread distribution of adipocytes throughout the body, strategies aimed at controlling weight gain and reducing excess adiposity may hold promise in mitigating the risk of cancer development. By targeting adipocyte biology and metabolism, interventions aimed at modulating adipose tissue function could potentially disrupt the pro-tumorigenic effects of adipocytes and enhance anti-cancer immune responses.

In summary, understanding the intricate crosstalk between adipocytes and T cells in the TME is essential for unraveling the mechanisms driving cancer progression and developing effective therapeutic strategies. Continued research efforts in this field will be crucial for identifying novel targets for cancer immunotherapy and improving clinical outcomes for cancer patients.

## Footnotes

1 VLCAD (very-long-chain acyl-CoA dehydrogenase); *Cpt1a* (carnitine palmitoyltransferase 1A); *FAO* (fatty acid oxidation); PD-1 (programmed cell death protein 1); SIRT1 (sirtuin 1)

2 Cpt1a (carnitine palmitoyltransferase 1A)
